# An investigation Into Traditional Chinese Medicine Hospitals in China: Development Trend and Medical Service Innovation

**DOI:** 10.15171/ijhpm.2016.72

**Published:** 2016-06-07

**Authors:** Liang Wang, Sizhuo Suo, Jian Li, Yuanjia Hu, Peng Li, Yitao Wang, Hao Hu

**Affiliations:** ^1^State Key Laboratory of Quality Research in Chinese Medicine, Institute of Chinese Medical Sciences, University of Macau, Taipa, Macau.; ^2^Faculty of Arts and Humanity, University of Macau, Taipa, Macau.

**Keywords:** Traditional Chinese Medicine (TCM) Hospital, Western Medicine (WM), Traditional Chinese Medicine (TCM), Medical Service, China

## Abstract

**Background:** This paper aims to investigate the development trend of traditional Chinese medicine (TCM) hospitals in China and explore their medical service innovations, with special reference to the changing co-existence with western medicine (WM) at TCM hospitals.

**Methods:** Quantitative data at macro level was collected from official databases of China Health Statistical Yearbook and Extracts of Traditional Chinese Medicine Statistics. Qualitative data at micro level was gathered through interviews and second-hand material collection at two of the top-level TCM hospitals.

**Results:** In both outpatient and inpatient sectors of TCM hospitals, drug fees accounted for the biggest part of hospital revenue. Application of WM medical exanimation increased in both outpatient and inpatient services. Even though the demand for WM drugs was much higher in inpatient care, TCM drugs was the winner in the outpatient. Also qualitative evidence showed that TCM dominated the outpatient hospital service with WM incorporated in the assisting role. However, it was in the inpatient medical care that WM prevailed over TCM which was mostly applied to the rehabilitation of patients.

**Conclusion:** By drawing on WM while keeping it active in supporting and strengthening the TCM operation in the TCM hospital, the current system accommodates the overriding objective which is for TCM to evolve into a fully informed and more viable medical field.

## Background

Traditional Chinese medicine (TCM) is a sector composed of TCM service (by TCM doctors) and TCM products (including Chinese patent medicine, Chinese herbal piece, and Chinese herb). TCM hospitals are medical institutions that treat the patients with TCM service and products to maintain public health.^1^ Before the founding of the People’s Republic of China in 1949, there were no established TCM hospitals, but only private pharmacies operated by TCM doctors.^2^ To protect TCM as a national treasure of China and provide affordable medical services, the central government initiated a TCM hospital system since 1954.^3^ Since then, TCM hospitals in China have remained committed to not only medical care but also the social and historic cause of reviving TCM as an important part of Chinese cultural heritage.^4^

Despite the interruption of the Cultural Revolution in the late 1960s and 1970s TCM still made some progress, such as acupuncture anesthesia. Moreover, “integration of Chinese and western medicine (WM)” was advocated by government to encourage TCM and WM practitioners to learn from each other to improve their medical practices. With the country’s opening to the outside world since 1978, more and more modern medical discoveries, practices and technologies from the West have been introduced to China, posing even greater challenges to TCM than ever before.^5^ Many TCM hospitals were obliged to adopt more and more WM in outpatient prescription and inpatient treatment.^6^ WM drugs, including chemical and biopharmaceutical drugs, have been applied in the TCM hospitals.^7^ As a consequence, TCM hospitals have begun to lose their distinct status in China’s medical sector.

Facing the challenges of WM and their diminishing identity as an independent medical body, could TCM hospitals orient themselves to a new way of developing their business? This question has drawn close social and professional attention and spurred heated debates. Some advocates, mostly TCM doctors, hold the view that TCM hospitals should opt for a purist model characterized by relying on TCM medical staff and TCM treatment only without WM medication and equipment.^8,9^ But others insist that TCM hospitals should transform themselves into a general hospital model, with TCM being just a department of the hospital while WM playing the leading role.^10^ Outside these opposing views, some people believe that TCM hospital should keep TCM as the mainstay and maintain a working alliance with WM.^11-13^

The debates have continued to linger on over the past decades, but there is still lack of adequate empirical evidence of the development trend, nor systematic information about medical services, as far as TCM hospitals are concerned. While modernisation of TCM products has attracted much attention, attention on TCM hospitals is comparatively limited.^14^ In particular, it remains indefinite how TCM and WM at TCM hospitals have interacted with each other in the past decades, and the issue of how to innovate medical services therein under these two diverse and distinctive medical knowledge systems is worth further exploration. As medical innovation plays influential role in reshaping medical sector and changing pharmaceutical system,^15^ exploratory investigation into TCM hospitals is especially necessary.

It is against this background that the present study aims at an informed analysis of the development trend of TCM hospitals in China and the situation of their medical service innovation, particularly, in relation to WM. To meet this research objective, data analysis at both macro and micro levels were implemented to ensure a breadth of knowledge appropriate to our purpose.

## Methods

Integrated quantitative and qualitative methodologies were used to obtain comprehensive and significant result of the innovative changes at TCM hospitals for an informed anticipation of their development trend. Statistical information was collected from official databases to take the long view at macro level. On the other hand, qualitative analysis of interviews conducted in two prominent TCM hospitals was employed to affirm the data result at micro level.

### Quantitative Investigation

The quantitative data was collected from two main sources. Firstly, the general trend data of TCM hospitals in China was collected from the *China Health Statistical Yearbook* database, officially gathered and publicized by the China Ministry of Health on a yearly basis. With sustained annual accumulation, *China Health Statistical Yearbook* has been recognized as a reliable source of medical information in China. From the yearbook database, two types of data were extracted: number of TCM hospitals and number of hospital beds. To reflect the historical development trend of TCM hospitals in China, the data collected covers the period from 1950 to 2013.

Secondly, the overall data about medical services of TCM hospitals were collected from the database of *Extracts of Traditional Chinese Medicine Statistics*, which is an official collection of TCM information operated by the State Administration of Traditional Chinese Medicine (SATCM). Through this database, data specific to the public TCM hospitals during the period between 2002 and 2013 were extracted in four aspects: (1) outpatient medical fees (including registration fee, drug fee, examination fee, and treatment fee); (2) inpatient medical fees (including sickbed fee, drug fee, examination fee, treatment fee, and surgery fee)^ [1] ^; (3) TCM and WM drug revenue; and (4) certified TCM staffs (doctors, assistant doctors, and pharmacists). Specifically, the composition of outpatient fees, inpatient fees, and TCM and WM drug revenue was analyzed to reflect the changes of TCM and WM medical services.

### Qualitative Investigation

To deepen the understanding of medical service innovation at TCM hospitals, qualitative investigation was cocurrently conducted. Chengdu TCM hospital and Jiangsu TCM hospital were meticulously chosen as typical cases for the investigation under the rationale that they are two of the earliest TCM hospitals in China established in 1950s as well as the most prestigious ones for their innovative achievements and the leadership role in the TCM society.

For data collection, semi-structured interviews were conducted at both hospitals. Three vice deans of Jiangsu TCM hospital and two vice deans and the head of Medical Service Department of Chengdu TCM hospital were interviewed. The interview covered mainly the following topics: medical service portfolio provided; the core medical service; medical service innovation at key departments; usage of TCM and WM drugs; and employment and cultivation of medical staffs (doctors and pharmacists), etc. For each topic, the interview began in an open-ended manner, gradually moving into more specific areas about how to deal with the coexistence between TCM and WM and how medical service was innovated. All the interviews were conducted in Chinese and taped. In addition to the interview, second-hand information about the two case TCM hospitals were also collected and referred to, including books on the hospital history, hospital leaflets, annual reports, and newspaper reports, etc.

For data analysis, a thematic analysis approach was applied. To ensure the validity of data analysis, two researchers did the thematic analysis separately at first. Then, the analysis results were compared to identify the similarities and the differences. Identified differences were analyzed by a third researcher to provide a triangular test. Finally, all the analysis results are combined to generate the final report on the qualitative investigation.

## Results

### Overall Development of Traditional Chinese Medicine Hospitals in China

**Figure 1:**
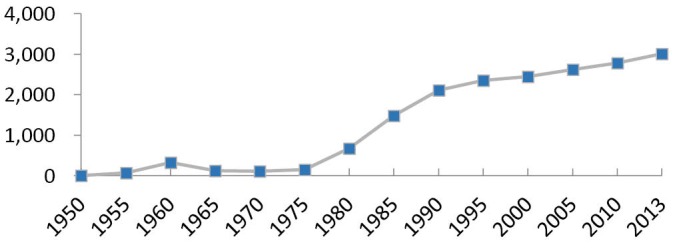
Quantity Development of Traditional Chinese Medicine (TCM) Hospitals in China (1950-2013).

The number of TCM hospitals increased slowly during the 1950-1975 period, but rose markedly after 1977. With only 184 in 1977, the TCM hospital figure climbed to 3015 in 2013, meaning that 93.9% TCM hospitals were set up after the Reform and Opening-up Policy of China (see Figure 1).

**Figure 2:**
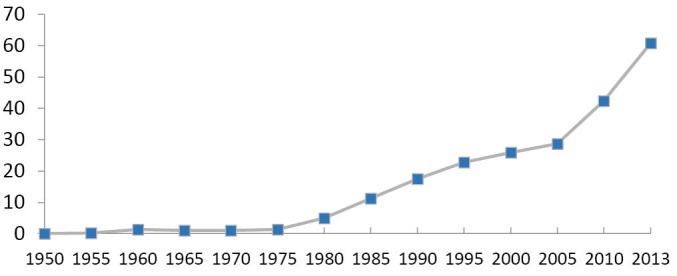
Quantity Development of Sickbeds in Traditional Chinese Medicine (TCM) Hospitals (1950-2013) (Unit: 10 000 Sickbeds).

Similar to the quantity hike of TCM hospitals, the number of sickbeds therein has achieved a rapid growth since 1977. Especially after 2005, the sickbed number increased dramatically and surpassed the growth rate of TCM hospitals, indicating that TCM hospitals attached more importance to expanding the hospital scale rather than the number in recent years (see Figure 2).

### Analysis of Medical Fees at the Outpatient

As shown in Table 1, the average outpatient medical fee/person-time at public TCM hospitals has increased about three times during the 2002-2013 period, out of which drug fees accounted for more than 60%. Specifically, the proportion of the examination fee has exceeded that of the treatment fee since 2003, showing increasing usage of instrumental WM examination at outpatient hospital care.

**Table 1:** Outpatient Medical Fee/Person-Time at Public TCM Hospitals (2002-2013) (Unit: RMB)

**Year**	**Outpatient Medical Fee**	**Registration Fee (%)**	**Drug Fee (%)**	**Examination Fee (%)**	**Treatment Fee (%)**
2002	69.5	1.3 (1.9)	44.0 (63.3)	7.8 (11.2)	8.0 (11.5)
2003	78.1	1.2 (1.5)	49.0 (62.7)	9.4 (12.0)	8.9 (11.4)
2004	83.0	1.3 (1.6)	49.4 (59.5)	11.2 (13.5)	9.8 (11.8)
2005	92.8	1.3 (1.4)	54.9 (59.2)	12.9 (13.9)	11.0 (11.9)
2006	95.6	1.3 (1.4)	55.6 (58.2)	13.8 (14.4)	11.7 (12.2)
2007	101.3	1.4 (1.4)	58.6 (57.8)	15.4 (15.2)	11.9 (11.7)
2008	113.4	1.4 (1.2)	66.5 (58.6)	17.2 (15.2)	12.9 (11.4)
2009	123.9	1.5 (1.2)	73.3 (59.2)	18.6 (15.0)	13.9 (11.2)
2010	137.5	1.5 (1.1)	82.6 (60.1)	20.3 (14.8)	15.0 (10.9)
2011	152.9	1.9 (1.2)	93.4 (61.1)	22.0 (14.4)	15.9 (10.4)
2012	166.1	2.0 (1.2)	101.8 (61.3)	23.9 (14.4)	15.7 (9.5)
2013	182.1	2.0 (1.1)	118.5 (65.1)	25.9 (14.2)	17.4 (9.6)
Growth rate	9.2%	4.1%	9.4%	11.5%	7.3%

Abbreviation: TCM, traditional Chinese medicine.

### Analysis of Medical Fees at the Inpatient

As depicted in Table 2, the inpatient medical fee is much higher than that of the outpatient, suggesting that most of the income of TCM hospitals comes from the inpatient medical service rather than the outpatient. From 2002 to 2013, the average inpatient medical fee had increased more than twice, with an average yearly growth rate of 7.8%. Of the total inpatient medical fee, the drug fee accounted for 42%-47%, implying that TCM hospitals are still dependent on drug for profit. In addition, while the proportion of treatment fee and surgery fee has decreased, the proportion of examination fee kept increasing, with an average yearly growth rate of 11.1%, indicating the rising usage of instrumental WM examination in inpatient service at TCM hospitals.

**Table 2:** Inpatient Medical Fee/Person at Public TCM Hospitals (2002-2013) (Unit: RMB)

**Year**	**Inpatient Medical Fee**	**Sickbed Fee (%)**	**Drug Fee (%)**	**Examination Fee (%)**	**Treatment Fee (%)**	**Surgery Fee (%)**
2002	2596	178 (6.9)	1225 (47.2)	137 (5.3)	530 (20.4)	202 (7.8)
2003	2854	185 (6.5)	1343 (47.1)	150 (5.3)	562 (19.7)	233 (8.2)
2004	3125	200 (6.4)	1430 (45.8)	176 (5.6)	626 (20.0)	275 (8.8)
2005	3520	223 (6.3)	1603 (45.5)	200 (5.7)	677 (19.2)	339 (9.6)
2006	3462	224 (6.5)	1536 (44.4)	204 (5.9)	651 (18.8)	363 (10.5)
2007	3720	233 (6.3)	1668 (44.8)	230 (6.2)	668 (18.0)	368 (9.9)
2008	4087	236 (5.8)	1877 (45.9)	253 (6.2)	738 (18.1)	379 (9.3)
2009	4417	235 (5.3)	2047 (46.3)	282 (6.4)	805 (18.2)	378 (8.6)
2010	4878	248 (5.1)	2261 (46.4)	317 (6.5)	888 (18.2)	398 (8.2)
2011	5206	253 (4.9)	2372 (45.6)	349 (6.7)	973 (18.7)	397 (7.6)
2012	5483	252 (4.6)	2458 (44.8)	381 (6.9)	857 (15.6)	348 (6.3)
2013	5917	267 (4.5)	2519 (42.6)	435 (7.4)	953 (16.1)	349 (5.9)
Growth rate	7.8%	3.8%	6.8%	11.1%	5.5%	5.1%

Abbreviation: TCM, traditional Chinese medicine.

### Analysis of Traditional Chinese Medicine and Western Medicine Drug Revenue

Table 3 shows that the average drug revenue of TCM hospitals during 2012-2013 has increased about six times. WM drug accounted for about 60% of the total drug revenue, more than TCM drug. But TCM drug income has a faster annual growth rate (20.2%).

**Table 3:** Average Drug Revenue/Hospital at Public TCM Hospitals (2002-2013) (Unit: 1000 RMB)

	**Total Drug Revenue**		**Outpatient Drug Revenue**		**Inpatient Drug Revenue**	
	**WM (%)**	**TCM (%)**	**WM (%)**	**TCM (%)**	**WM (%)**	**TCM (%)**
2002	3819 (62.8)	2259 (37.2)	1928 (51.0)	1853 (49.0)	1891 (82.3)	406 (17.7)
2003	4421 (64.6)	2421 (35.4)	2162 (51.9)	2001 (48.1)	2259 (84.4)	419 (15.7)
2004	5136 (67.6)	2463 (32.4)	2392 (54.5)	1997 (45.5)	2744 (85.5)	466 (14.5)
2005	6059 (67.0)	2983 (33.0)	2725 (53.4)	2380 (46.6)	3334 (84.7)	603 (15.3)
2006	6567 (67.2)	3213 (32.9)	2924 (52.8)	2609 (47.2)	3643 (85.8)	604 (14.2)
2007	8204 (67.7)	3915 (32.3)	3462 (52.7)	3113 (47.3)	4742 (85.5)	802 (14.5)
2008	10 494 (67.4)	5085 (32.6)	4212 (51.5)	3961 (48.5)	6282 (84.8)	1124 (15.2)
2009	12 774 (66.4)	6462 (33.6)	4886 (49.7)	4945 (50.3)	7888 (83.9)	1517 (16.1)
2010	15 190 (64.7)	8279 (35.3)	5609 (47.1)	6289 (52.9)	9581 (82.8)	1990 (17.2)
2011	17 781 (61.5)	11 153 (38.6)	6450 (43.4)	8424 (56.6)	11 331 (80.6)	2729 (19.4)
2012	21 164 (59.2)	14 562 (40.8)	7337 (40.6)	10 746 (59.4)	13 827 (78.4)	3817 (21.6)
2013	23 500 (57.9)	17 115 (42.1)	8151 (39.3)	12 608 (60.7)	15 349 (77.3)	4507 (22.7)
Growth rate	18.0%	20.2%	14.0%	19.0%	21.0%	24.5%

Abbreviations: TCM, traditional Chinese medicine; WM, western medicine.

For outpatient service, the average annual growth rate of TCM drug was 19%, higher than that of WM drug which was 14%. From the beginning of 2004, the proportion of TCM drug income kept increasing, exceeded WM in 2009 and reached 60.7% in 2013.

Conversely, for inpatient service, WM drug accounted for about 80% of the total inpatient drug revenue. Even so, the average annual growth rate of TCM drug income reached 24.5%, higher than that of WM drug which was 21%.

### Medical Staff at Traditional Chinese Medicine Hospitals

With increasingly greater number and scale of public TCM hospitals, the total number of doctors increased at an average rate of 5.3% per year, while that of the TCM doctors was only 4.6% (see Table 4). As of 2013 only 50.2% doctors in public TCM hospitals were certified TCM doctors, whereas the proportion of TCM assistant doctors took up only 20%-30%. For pharmacists, the number of TCM pharmacists grew at a faster yearly rate than that of the total pharmacists, accounting for 56% of them in 2013.

**Table 4:** Medical Staff at Public TCM Hospitals (2002-2013) (Unit: Person)

	**Doctors**		**Assistant Doctors**		**Pharmacists**	
	**WM (%)**	**TCM (%)**	**WM (%)**	**TCM (%)**	**WM (%)**	**TCM (%)**
2002	52 648 (46.3)	61 136 (53.7)	13 621 (69.2)	6055 (30.8)	-	-
2003	57 369 (48.4)	61 159 (51.6)	14 319 (71.3)	5751 (28.7)	-	-
2004	60 843 (49.8)	61 286 (50.2)	14 766 (72.8)	5525 (27.2)	-	-
2005	63 323 (50.7)	61 553 (49.3)	15 008 (73.9)	5286 (26.1)	-	-
2006	68 079 (52.0)	62 773 (48.0)	15 064 (73.7)	5377 (26.3)	-	-
2007	70 990 (51.6)	66 529 (48.4)	14 271 (74.8)	4813 (25.2)	19 136 (48.2)	20 543 (51.8)
2008	75 340 (52.1)	69 233 (47.9)	14 106 (75.4)	4606 (24.6)	18 430 (45.9)	21 722 (54.1)
2009	80 720 (51.9)	74 887 (48.1)	14 179 (75.1)	4696 (24.9)	18 282 (44.5)	22 830 (55.5)
2010	84 967 (51.3)	80 519 (48.7)	14 620 (75.7)	4692 (24.3)	19 030 (44.4)	23 809 (55.6)
2011	89 744 (52.1)	82 508 (47.9)	13 738 (74.3)	4742 (25.7)	19 993 (44.5)	24 927 (55.5)
2012	94 269 (50.4)	92 678 (49.6)	13 494 (71.2)	5457 (28.8)	20 572 (43.8)	26 352 (56.2)
2013	100 089 (49.8)	100 726 (50.2)	13 439 (69.5)	5894 (30.5)	21 864 (44.0)	27 801 (56.0)
Growth rate	6.0%	4.6%	-0.1%	-0.2%	2.2%	5.2%

Abbreviations: TCM, traditional Chinese medicine; WM, western medicine.

### Findings From the Qualitative Analysis

Apart from the statistical data described above, the qualitative investigation also generated meaningful information pertinent to the outpatient and inpatient services, the TCM and WM drug usage, and the TCM medical staff development.

*TCM Predominating the Outpatient: TCM Diagnosis With WM Examination. In the outpatient departments of TCM hospitals, most departments used TCM medical treatment. The hospitals in our case study took advantage of the characteristic features of TCM to draw more patients. One of the interviewees described the situation as below:*

*“As a TCM hospital, our main advantage or main business comes from our featured TCM outpatient service, using featured TCM treatment and drugs to serve patients….In terms of reliance on TCM, 70% of all the outpatient cases has used or involved TCM treatment. For some TCM specialists, the percentage of TCM application could go beyond 80% even 90%”* (Interviewee of Jiangsu A).

WM was mainly applied in medical examination through using modern WM devices in support of the diagnosis of TCM. In general practice, TCM doctors made a preliminary diagnosis based on the TCM theory first, then the WM examination result was used to confirm or modify their diagnosis. As informed in one of our interviews:

*“Most of our hospital profit comes from the fee charged on drug, surgery, and examination, among which examination accounted for the biggest revenue. Examination includes medical filming, type-B ultrasonic checking, and body testing, etc. In the end, it is mainly TCM that provides diagnosis and prescription”* (Interviewee of Jiangsu B).

In outpatient treatment, the TCM hospitals have followed the principle of TCM-over-WM priority, ie, “whenever TCM is therapeutically viable, do not use WM; and where necessary, integrate WM into TCM,” in line with the overriding objective of “consolidating the TCM characterized by benefiting from the assistance of WM.”

*WM predominating the inpatient: WM surgery with TCM for rehabilitation.* By contrast, WM contributed more in the inpatient treatment. In the inpatient departments of TCM hospitals, WM rather than TCM was mostly applied to treat patients and brought financial support to the hospital as well. Especially, WM surgery was applied to treat more urgent cases in the inpatient, whereas TCM inpatient services are usually used as non-communicable disease management or pains. As acknowledged by the interviewees:

*“WM is mainly used in inpatient departments. For example, to perform a surgery, we have advanced instruments and techniques. But to recover after the surgery, we will incorporate TCM to optimize the patient care for better recovery”* (Interviewee Jiangsu A).

*“The inpatient of TCM hospital emphasizes both TCM and WM. To some extent, we emphasize more on WM, like surgeries, etc. But before and after the surgery we will apply TCM drugs”* (Interviewee Jiangsu B).

*Relying on WM drugs while innovating TCM drugs.* The interviewees admitted that as a major part of hospital income WM drug was used more than TCM in their hospitals. However, to highlight the special characteristics of a TCM hospital, they also made an effort to recreate TCM drugs, including Chinese herbal pieces, Chinese proprietary medicine, and hospital preparations. As described by one of the interviewees:

*“We have used the Chinese herbal piece exclusively with small packaging, which helps to improve drug quality and patients’ rights to know about their treatment. Moreover, in recent years, we have invested in R&D and improved dozens of hospital preparations formulas, processing techniques, quality standards and packaging, which significantly upgraded the internal quality and external image of TCM drugs”* (Interviewee Chengdu B).

*Cultivating the TCM medical staff.* The TCM hospitals under study believed that the unique features of TCM hospital care could not be maintained without qualified staff knowledgeable in TCM. The hospital leadership insisted on using mentorship system to train TCM doctors even if they had got a degree from TCM universities. As one put it:

*“Our hospital strengthened our cultivation of TCM doctors by innovating our staff development mechanism. For example, we have founded the Experience Sharing Workshops by veteran TCM doctors. We also required new TCM doctors to follow senior TCM doctors in clinical services to acquire their TCM experience and knowledge”* (Interviewee Jiangsu C).

The directors being interviewed hold that such in-house training system could facilitate maintaining TCM traditions and also benefit the TCM innovation. As clearly expressed by one of them:

*“Most of the current TCM doctors have learned WM. So they could integrate traditional theory of TCM with anatomy, modern pharmacology and physiology of WM, which differentiated themselves from conventional TCM doctors. In clinical service, they could combine TCM and WM to realize the modernization of TCM”* (Interviewee Jiangsu A).

On the other hand, the TCM hospitals in question tried to cultivate the needed TCM knowledge and skills of the WM doctors. In both of the hospitals, the WM doctors were required to learn TCM if they had never had TCM education background before.

## Discussion

Shown in this study, TCM hospitals were transforming themselves from past small and specialized medical institutions to comprehensive and diversified medical service providers.^16,17^ As such, TCM hospitals are compelled to innovate to meet the rising demands of patients and the competition pressures from general hospitals that apply WM in the main. As our quantitative and qualitative data showed, all the TCM hospitals in China today were providing both TCM and WM medical services to the outpatient and the inpatient as well. There are several reasons for such kind of change. Firstly, the political impact of the “integrating medical services of both TCM and WM” policy in 1970s brought WM into TCM hospital system mandatorily.^18,19^ Later on more and continuing policies were issued to boost the integration of WM into TCM. For example, the “*Basic Standards for Medical Institutions*” publicized by the Ministry of Health in 1994 explicitly required first-class TCM hospital to be equipped with adequate WM instruments.^20^ Secondly, the transformation from planning economy to market economy brought market pressures to TCM hospitals for utilizing WM. In the traditional planning economy, all the expense of TCM hospitals were covered by the government.^21^ However, the transformation to market economy forced hospitals to compete with each other.^22^ While government still supplied financial supports to some extent, it could not afford the whole expense of TCM hospitals.^23^ Moreover, TCM medical service on the whole costs less than their WM counterpart, and therefore, brings insufficient financial resources for TCM hospital. For survival reasons, TCM hospitals had to bring in WM to provide more profitable medical services, such as medical examination, etc.^24,25^ Thirdly, patients were getting impatient with most of the traditional way of TCM and requesting more advanced medical services.^26^ Lastly, the TCM practitioners themselves also had motivation to learn from WM. They generally recognized the limitations of TCM in some aspects and hoped that the WM knowledge and technologies could benefit their practice of TCM.^27^ All the factors collectively pushed TCM hospitals into providing TCM and WM medical services simultaneously.

Our data showed that, while the situation necessitated TCM hospitals’ inclusion of WM into their medical service, they were still trying to maintain their special TCM features. WM medical services were mostly conducted in the inpatient, or the outpatient departments where surgery or immediate medical treatment was called for. A case in point of the most welcome WM presence in TCM was medical examinations. In both of outpatient and inpatient services, instances of using WM in medical examination kept rising. However, medical examination was only used to support the diagnosis made by TCM doctors in the outpatient, where, most notably, TCM medical services still predominated. Doctors diagnosed illness and prescribed treatment by following TCM creed and techniques, which was still upheld firmly by TCM hospitals. On the one hand, such practice reflected the apprehension of TCM hospitals about losing the professional legitimacy and the government recognition, particularly, legal and financial support.^28^ In addition, it suggested that TCM hospitals were aware of using TCM medical service as its competitive edge to win over Chinese patients with strong belief in and preference for TCM.^29^

Due to the shortage of fiscal supply, TCM hospitals opted to rely on the revenue from their drug usage to cover their operational expenses. Compared with the more expensive and profitable WM drugs, TCM drugs brings less income.^30^ Despite the disadvantage, as our data showed, TCM hospitals tried not only to improve TCM drugs to generate more revenue but also to strengthen the hallmark of TCM on their products. These undertakings were evidenced by their effort to improve Chinese herbal pieces and hospital preparations, a prime example being the production and packaging of Chinese herbal pieces in cooperation with good manufacturing practice (GMP) known as new GMP of WM drugs, which not only increased profits but earned more credit from the quality-minded customers.

This study also revealed the challenge to the qualification of the TCM staff. *SATCM* in 2007 introduced the “Guide for evaluating TCM characteristics of TCM hospital,” requiring that certified TCM staff (doctors and pharmacists) must account for more than 60% of total medical staff in TCM hospitals. But our data showed the current percentage was still below the government requirement, meaning there was a shortage of qualified TCM medical staff. Apart from the rapid expansion in hospital scale, there are several reasons for the shortage. First, TCM universities now train students for TCM doctors through a more WM-oriented system, overemphasizing WM knowledge and skills at the expense of TCM.^31^ Second, the current TCM certification tests rests heavily on acquisition of WM knowledge, which is not easy for the applicants who were trained as TCM professionals.^32^ Therefore, it was deemed imperative for TCM hospitals to establish their own training and certifying systems to cultivate medical staff who were experts on both TCM and WM through mentorship teaching to develop TCM medical staff even if they had got university degrees, as well as improving the maintenance and innovation of TCM medical services.^33,34^

At the same time, it must realize that there are still challenges for TCM hospitals ahead. First, the challenges of TCM product quality are affecting the medical service of TCM hospitals. In the past years, especially the quality challenges of Chinese herbal piece has significantly influenced the medical effects of TCM service, which raised much public concerns and complaints on medical service at TCM hospital. Second, while government provides many supports to TCM hospitals, it is increasing its expectations on TCM hospitals. It is obvious that government at different levels are requiring more significant improvement from TCM hospitals, which has raised much pressure on TCM hospitals. How to respond to such kind of rising expectation will become a main challenge in the near future.

Form this study, management implications for different parties could also be provided. For TCM hospitals, they should refer to WM logic to reinterpret their own TCM logic to establish their competitive advantages. Appropriate combination of two kinds of medical logics will bring much innovation for TCM medical service. For general hospitals, they can also learn from the innovative practices of TCM hospitals to enrich their medical service. Considering their comparative advantages in medical equipment and facilities, if general would like to introduce the innovative treatment from TCM hospitals, it is obvious that general hospitals will be able to apply these practices to a broader context.

There are several limitations that could be resolved in future studies. First, this study found out the obvious differences between TCM and WM with respect to the inpatient as opposed to outpatient, which necessitates a further study on the interaction between TCM and WM in single therapeutic areas to deepen the understanding of the disparity. Second, this study has not addressed the relationship between financial stability and medical service innovation. As financial factors have become influential determinants for portfolios of TCM and WM medical service, further exploration into the impact of financial factors on TCM medical service innovation at TCM hospitals needs to be conducted.

## Conclusion

TCM hospitals in China maintained as a unique and popular medical care provider through offering both TCM and WM services and strived to incorporate WM to support and strengthen TCM. In particular, they opted to blend WM with TCM to recreate a new type of TCM medical service. The future survival and development of TCM hospitals are contingent on to what extent they could maintain the traditional characteristics of TCM and whether its medical innovation could be realized concurrently.

## Acknowledgements

The authors would like to express their sincere thanks to the funding support of University of Macau, Taipa, Macau (MYRG2015-00072-ICMS-QRCM).

## Ethical issues

The research is ethically approved by the University of Macau, Taipa, Macao.

## Competing interests

Authors declare that they have no competing interests.

## Authors’ contributions

LW, SZS, HH, and YTW designed the study. LW and SZS conducted data collection and analysis. LW, SZS, HH, JL, YH, and PL drafted the manuscript. All the authors reviewed and approved the whole manuscript. LW and SZS contributed as joint first researchers.

## Authors’ affiliations

^1^State Key Laboratory of Quality Research in Chinese Medicine, Institute of Chinese Medical Sciences, University of Macau, Taipa, Macau. ^2^Faculty of Arts and Humanity, University of Macau, Taipa, Macau.

## Endnote


^[1]^ *Examination fee*: It means any fee charged by hospital for providing medical test services to patients, such as blood test, ultrasonic inspection, etc, which only includes western medicine. *Treatment fee*: It means any fee charged by hospital for providing direct but non-surgery medical treatment to patients, such as having an injection, physical therapy, acupuncture, etc, which can include both TCM and western medicine. *Surgery fee*: It means any fee charged by hospital for providing direct surgery service to patient, which only includes western medicine.


## Key messages

**Implications for policy makers**

- Traditional Chinese medicine (TCM) medical service on the whole costs less than their western medicine (WM) counterpart, and therefore, brings insufficient financial resources for TCM hospital.
- Despite the disadvantage, TCM hospitals tried not only to improve TCM drugs to generate more revenue but also to strengthen the hallmark of TCM on their products.

**Implications for public**

In the inpatient departments of traditional Chinese medicine (TCM) hospitals, western medicine (WM) rather than TCM was mostly applied to treat patients and brought financial support to the hospital as well. Especially, WM surgery was applied to treat more urgent cases in the inpatient, whereas TCM was mainly used to facilitate the patient’s recovery from surgery.
